# Surgical management of ameloblastoma. Review of literature

**DOI:** 10.4317/jced.55452

**Published:** 2019-01-01

**Authors:** David Neagu, Oscar Escuder-de la Torre, Inés Vázquez-Mahía, Nicolás Carral-Roura, Guillermo Rubín-Roger, Ángel Penedo-Vázquez, Ramón Luaces-Rey, José-Luis López-Cedrún

**Affiliations:** 1 MD. Maxillofacial Surgery Department, University Hospital A Coruña, Spain; 2 MD. Head of Maxillofacial Surgery Department, Hospital Parc Tauli Sabadell, Spain; 3 MD, PhD. Maxillofacial Surgery Department, University Hospital A Coruña, Spain; 4 MD. Maxillofacial Surgery Department, University Hospital A Coruña, Spain; 5 MD, DDS, PhD. Head of Maxillofacial Surgery Department, University Hospital A Coruña, Spain

## Abstract

**Background:**

Ameloblastoma is an odontogenic tumor that represents 1% of all tumors in the oral cavity and it is clinically classified in three types. Currently, solid and multi-cystic are considered locally aggressive, with high recurrence rates with conservative treatment.

**Material and Methods:**

Objective of the present review is to assess whether the surgical treatment should be conservative or radical. English articles published between 2009-2014, with available summary and in humans were included.

**Results:**

241 articles were found, 188 were excluded because analyzing. 53 articles were analyzed and finally 14 were selected for this review.

**Conclusions:**

The optimal surgical treatment of ameloblastoma should minimize recurrences, restore function and aesthetic and present a minimal morbidity in the donor area. Surgical planning must be performed based on the patient comorbidities, the size and location of the tumor, the techniques available for reconstruction and the surgeon’s experience-Radical surgery appears to be the most recommended option in multicystic / solid and advanced unicystic tumors, along with long-term follow-up for the possibility of recurrence beyond 10 year. Conservative surgery combined with a support technique and long-term follow-up is reserved for the unicystic and multicystic / solid types if small extension. Prospective and randomized studies for ameloblastoma are recommended.

** Key words:**Ameloblastoma, surgery, enucleation, radical.

## Introduction

Ameloblastoma is an odontogenic tumor that represents 1% of all tumors fin the oral cavity, with an incidence of 0.5 per million inhabitants per year (1,2). It is a benign tumor, which usually occurs between the third and fifth decades, with the same frequency in men and women, and the location is 80% - 20% in the jaw and maxillary respectively (3,4).

The origin can be the embryonic remains of odontogenic cysts, the dental sheet, the enamel organ or the stratified squamous epithelium of the oral cavity. The pathogenesis is unknown. Different mechanisms such as inflammation, chronic trauma, malnutrition, vitamin deficiency, as well as a possible relationship with HPV have been described as triggers of the process (5).

It is a slow growth tumor that rarely gives metastasis. It can cause destruction of the cortical bone. It causes invasion of the surrounding soft tissue, producing pain, asymmetry, speech and agglutination, malocclusion, loss of dental pieces, and paresthesia if the lower alveolar nerve is affected (1,3).

Diagnosis is usually done through orthopantomography incidentally or because patients consult for symptoms. Findings are not pathognomonic, and the lesion should be confirmed with a histological examination (3).

Mortality can be produced by invasion of vital structures, serious infections, recurrences or remote metastasis (4).

Ameloblastomas are clinically classified in three types: solid and multicystic, unicystic and peripheral. Currently, solid and multi-cystic are considered locally aggressive, with high recurrence rates with conservative treatment. Prognosis and surgical approach of both is similar (6).

On the other hand, unicystic ameloblastoma is less aggressive, with lower recurrence rate, and can respond better to conservative surgery (6).

Finally, the peripheral type may have another origin and responds well to local excision (6).

The elective treatment of ameloblastoma is surgery, but the application of conservative or radical techniques depending on the clinical type has always been controversial, especially in the solid / multicystic and unicystic. When talking about conservative surgery, we refer to enucleation or marsupialization, combined or not with support techniques such as curettage, Carnoy Solution or liquid nitrogen. In the case of radical surgery, the term refers to mandibulectomy or segmental resection of the lesion (7).

The items that should be considered when choosing the most appropriate surgical option are the recurrence rate, mortality and morbidity, functional recovery and aesthetic of the patient, as well as the quality of life after the treatment.

Objective of the present review is to assess whether the surgical treatment should be conservative or radical.

## Material and Methods

Data was collected using U.S. National Institutes of Health’s National Library of Medicine (NIH/NLM). Keywords surgical treatment ameloblastoma, surgical management ameloblastoma, radical surgery ameloblastoma, conservative surgery ameloblastoma were utilized. English articles published between 2009-2014, with available summary and in humans were included.

## Results

**Figure 1:**
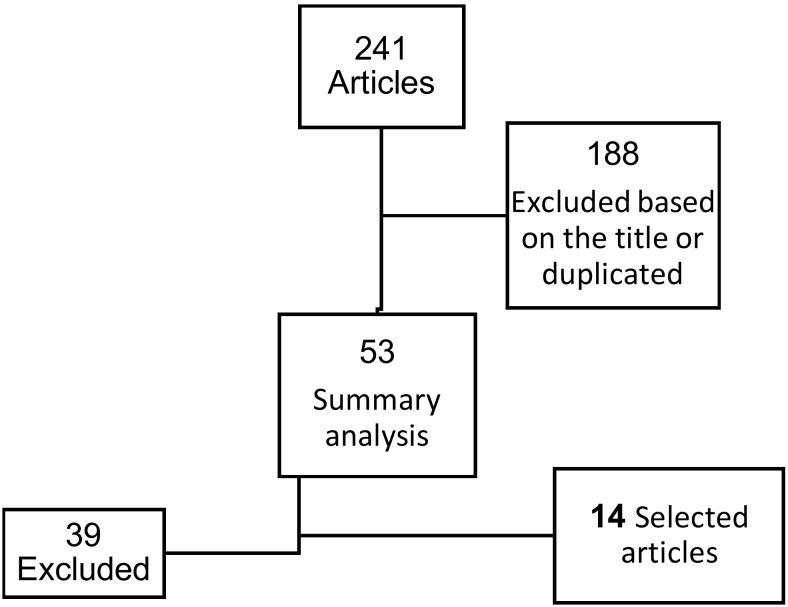
Flow chart referring article management.

241 articles were found, 188 were excluded because the title did not meet the objective of the present review or because they were repeated. The summary of the remaining 53 articles was analyzed and finally 14 were selected for this review, considering in most of them the impact factor of journals where they were published and excluding most of case reports (Fig. 1). Summary of review articles considering type of ameloblastoma, type of surgery proposed, and proposed follow up is exposed in  Table 1.

**Table 1:**
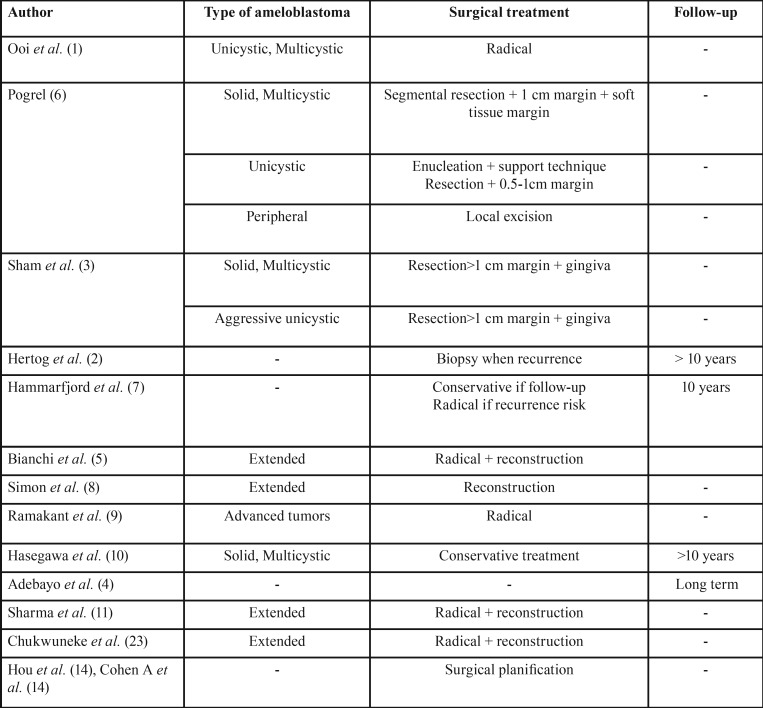
Summary of review articles considering type of ameloblastoma, type of surgery proposed, and proposed follow up.

Ooi *et al.* (1) publishes a retrospective review. Several parameters are considered when assessing the results of radical surgery in 30 patients with unicystic or multicystic ameloblastoma ( Table 2). The treatment was the segmental resection of the affected portion with subsequent reconstruction by means of a free peroneal flap. The segmental mandibulectomy treatment of unicystic and multicystic ameloblastoma showed no recurrence in a 5-year follow-up period, with acceptable aesthetic and functional results despite the low use of osteointegrated dental implants. Virtual surgical planning and other techniques could improve facial asymmetry and reduce the need for additional interventions.

**Table 2:**
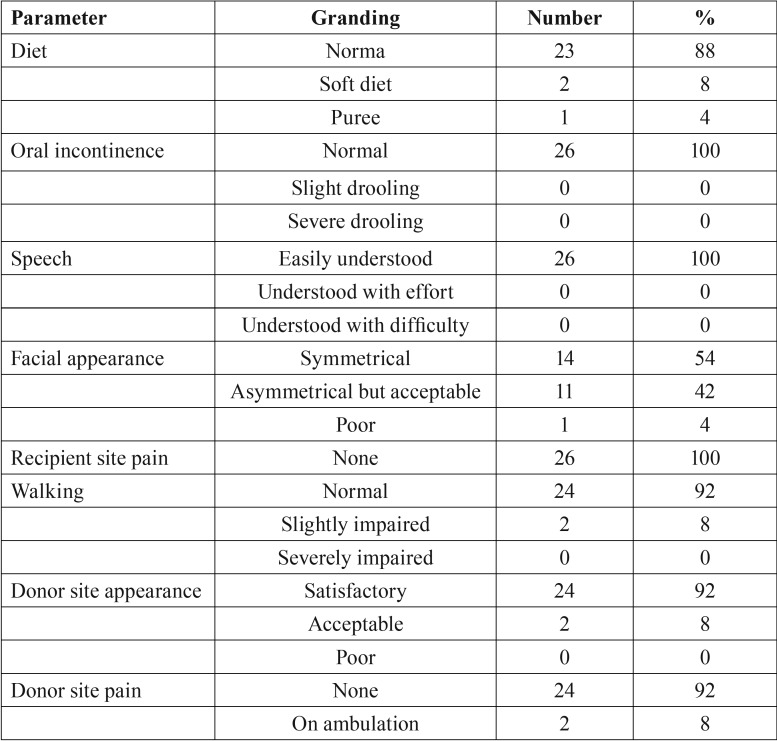
From Ooi A, Feng J, Tan HK, Ong YS. Primary treatment of mandibular ameloblastoma with segmental resection and free fibula reconstruction: Achieving satisfactory outcomes with low implantprosthetic rehabilitation. J Plast Reconstr Aesthet Surg. 2014 Apr; 67(4):498-5051.

Pogrel *et al.* (6) makes a review of 58 articles. No randomized clinical trials were available. The results showed a recurrence of 60-80% with simple enucleation of solid and multicystic ameloblastoma, therefore, the treatment should be the segmental resection with 1 cm of margin to the bone, including a soft tissue margin. For unicystic ameloblastoma, the authors recommend an enucleation accompanied by a support technique. If not possible, a block resection with 0.5-1 cm margins would be the correct technique. Finally, the peripheral ameloblastoma responds well to the local excision.

In the retrospective study of 6 patients and literature review developed by Sham *et al.* (3), it is recommended to make a resection with margins of at least 1 cm in the multicystic ameloblastoma and aggressive cases of the unicystic. Gingiva must be included. Immediate reconstruction must be done.

Hertog *et al.* (2) presents 18 cases of patients previously treated with enucleation and subsequently developed recurrences. Of these, 12 were treated with radical surgery without any recurrence, with a follow-up of 10.5 years. Relapse was present in all those cases who opted for conservative surgery. The authors recommend a biopsy before operating a possible recurrence and consider whether monitoring should take place beyond 10.5 years, due to the possible reappearance of the disease.

A retrospective study that analyzing the recurrences in 48 patients with an intrabony ameloblastoma is provide by Hammarfjord *et al.* (7). Radical treatment is recommended when recurrence and conservative option should be reserved for small intrabony ameloblastoma. However, given the low rate of metastases, the chosen technique must consider the quality of life and comorbidities of the patient. Thus, the authors suggest a conservative treatment of ameloblastoma along with an annual follow-up for 10 years as a good alternative to processes that would involve aggressive resections, especially if there are no medical and personal factors that could compromise follow-up. It seems that enucleation can be the best alternative when assessing all the important aspects for the patient, but radical surgery is the correct option when the lesion is close to vital structures or when it cannot be treated conservatively.

Bianchi *et al.* (5) likewise presents a retrospective study with 31 patients diagnosed with large ameloblastoma. The treatment of choice proposed by the authors for the tumors located in the mandibular branch should be the segmental resection followed by immediate reconstruction. This technique improves aesthetic and functional results and minimizes recurrences. Immediate dental implants should be considered an integral part of the treatment, especially in young patients.

The cohort study presented by Simon *et al.* (8) compares 32 patients treated radically for ameloblastoma and undergoing immediate reconstruction during the same surgical act with 32 patients treated for the same tumor but without any subsequent reconstruction. The patient who went reconstruction had better results in the surveys on the quality of life. On the other hand, in the group without reconstruction affectation of the speech and the eat was observed.

Ramakant *et al.* (9) makes a retrospective review comparing two groups of patients of 10 individuals each, treated radically or conservative. They consider that the conservative technique is not useful and involves more surgical interventions with a worse aesthetic and functional result. The treatment of choice according to this article is radical surgery, especially in advanced tumors.

The possibility of conservative treatment in the multicystic/ solid ameloblastoma is analyzed by the retrospective study done in 23 patients by Hasegawa *et al.* (10). An incidence of 48.7% of recurrences with conservative treatment was observed, less than documented until now in the multicystic/ solid variants. Consequently, for the authors conservative treatment can be used in the treatment of solid / multicystic ameloblastoma. Follow up by means of orthopantomography should be considered, even after 10 years postoperative.

Adebayo *et al.* (4) presents a patient treated with radical surgery that develops a soft tissue recurrence after 21 years. His recommendation is to carry out radiological follow-up throughout life due to the possibility of very late relapses.

When ameloblastoma is widespread, Sharma *et al.* (11) recommends radical surgery with reconstruction. Performing a mandibulectomy without reconstruction involves a high morbidity for the oral and facial function, as well as many psychological sequels (12).

Hou *et al.* (13) revision proposes an assisted CAD / CAM technique for surgery. It provides better view of the defect, decreases the surgical time, the loss of blood and the risk of ischemia of the flap. Models printed with 3D technology are an alternative of less cost to the previous technique and can be applied especially in small injuries (14).

## Discussion

The optimal surgical treatment of ameloblastoma should minimize recurrences, restore function and aesthetic and present a minimal morbidity in the donor area. Surgical planning must be performed based on the patient comorbidities, the size and location of the tumor, the techniques available for reconstruction and the surgeon’s experience.

When we talk about conservative technique, we refer to enucleation, curettage or marsupialization, which can be associated with cryotherapy with liquid nitrogen or tissue fixers like the Carnoy’s solution. It has a low morbidity and excellent aesthetic and functional results. The downside is the high rate of recurrences, which is between 60-80%6, especially if only simple enucleation is done.

Radical surgery implies marginal or segmental mandibulectomy with the need of 1-1.5 centimeters margins, since ameloblastoma cells can be found 8 mm apart from the radiological and clinical margin of the tumor (6,7). Restoring the functionality and aesthetics of the area in these cases can be a challenge. However, the low recurrence rate, around 0-10% 1, makes this technique a good option to avoid further interventions.

In the present review, if we refer to the type of tumor, the unicystic ameloblastoma is advisable to treat by means of enucleation and support technique. Good results are reported, despite the lack of long-term studies. Simple enucleation should not be used for the high recurrence rate6. If the tumor is aggressive or there is no possibility of combined conservative treatment, radical surgery with 0.5-1 cm of margins is advised with reconstruction, with acceptable outcomes (1,3,6).

In the case of solid and multicystic ameloblastoma the treatment recommended by most authors is a radical surgery with margins of 1 cm and resection of adjacent soft tissue with a subsequent reconstruction (1,3,6). Recurrence on soft tissue adjacent to ameloblastoma have been reported after 21 years post-surgery. The recurrence rate may be higher with this technique because of inadequate monitoring of patients (4). Some authors propose combined conservative surgery and long-term follow-up as a possible alternative, especially when there is good compliance by the patient and the risk of involvement of adjacent structures is low (7,10).

In the peripheral ameloblastoma the authors recommend local resection6.

When we take into consideration large tumors, the conservative technique has no utility and treatment should be radical. The reconstruction implies a higher score in quality assessment surveys, thanks to a faster speech recovery and functionality, a better aesthetic with less psychological problems and a lower morbidity for the patient (8,9,11,12). To describe the different techniques that exist for the post-surgical reconstruction of ameloblastoma is not the objective of this study, but it must be said that the virtual models currently available can be a support tool, offering better planning process (14).

## Conclusions

According to the present review, radical surgery appears to be the most recommended option in multicystic / solid and advanced unicystic tumors, along with long-term follow-up for the possibility of recurrence beyond 10 years. This treatment minimizes recurrences and reduces the need for new interventions. Currently, morbidity derived from reconstruction can be reduced by techniques supported by digital models, which are a useful tool to restore the aesthetics and functionality of the area, especially if they are complemented by osteointegrated dental implants.

Conservative surgery combined with a support technique and long-term follow-up is reserved for the unicystic and multicystic / solid types if small extension, although despite being less invasive, the recurrence rate is very high.

This review has the main limitation to be integrated by studies with a C level of evidence. Randomized and prospective studies are needed to determine the surgical treatment criteria, but low incidence of tumor is a limitation. Not all studies are consistent to evaluate the same items when treatments are compared.

For the future, prospective and randomized studies for ameloblastoma are recommended to define the surgical treatment criteria and design an algorithm for the management.
